# Machine Learning Classification of Self-Organized Surface Structures in Ultrashort-Pulse Laser Processing Based on Light Microscopic Images

**DOI:** 10.3390/mi15040491

**Published:** 2024-04-02

**Authors:** Robert Thomas, Erik Westphal, Georg Schnell, Hermann Seitz

**Affiliations:** 1 Chair of Microfluidics, Faculty of Mechanical Engineering and Marine Technology, University of Rostock, Justus-von-Liebig Weg 6, 18059 Rostock, Germany; robert.thomas@uni-rostock.de (R.T.); erik.westphal@uni-rostock.de (E.W.); 2 Department Life, Light & Matter, University of Rostock, Albert-Einstein-Str. 25, 18059 Rostock, Germany

**Keywords:** machine learning analysis, automated classification, nano- and microstructures, femtosecond laser, self-organized

## Abstract

In ultrashort-pulsed laser processing, surface modification is subject to complex laser and scanning parameter studies. In addition, quality assurance systems for monitoring surface modification are still lacking. Automated laser processing routines featuring machine learning (ML) can help overcome these limitations, but they are largely absent in the literature and still lack practical applications. This paper presents a new methodology for machine learning classification of self-organized surface structures based on light microscopic images. For this purpose, three application-relevant types of self-organized surface structures are fabricated using a 300 fs laser system on hot working tool steel and stainless-steel substrates. Optical images of the hot working tool steel substrates were used to learn a classification algorithm based on the open-source tool Teachable Machine from Google. The trained classification algorithm achieved very high accuracy in distinguishing the surface types for the hot working steel substrate learned on, as well as for surface structures on the stainless-steel substrate. In addition, the algorithm also achieved very high accuracy in classifying the images of a specific structure class captured at different optical magnifications. Thus, the methodology proposed represents a simple and robust automated classification of surface structures that can be used as a basis for further development of quality assurance systems, automated process parameter recommendation, and inline laser parameter control.

## 1. Introduction

In classical manufacturing, ultrashort-pulse laser (USPL) processing has become firmly established in recent decades, especially in drilling, cutting, and structuring processes, due to its nearly cold and residue-free material ablation [1,2,3]. With regard to surface modification, a wide variety of nano- and microscale surface structures can be produced in a one-step process at high fabrication speeds. In addition to laser-inscribed structures [4,5], of which the size corresponds to the laser focus diameter in the micro-range, the production of self-organized structures formed in the laser spot is particularly interesting.

Self-organized structures are formed by light–material interactions and after material ablation and solidification phenomena on the surface [6,7,8,9,10,11]. The resulting topography and morphology of these structures results from complex interactions of the laser light with the material properties [12,13,14], the scanning strategy [12,15], and the environmental conditions [16]. According to their morphology and topography, the self-organized structures can be divided, for example, into laser-induced periodic surface structures (LIPSSs), craters, and microstructures [12].

**Figure 1:**
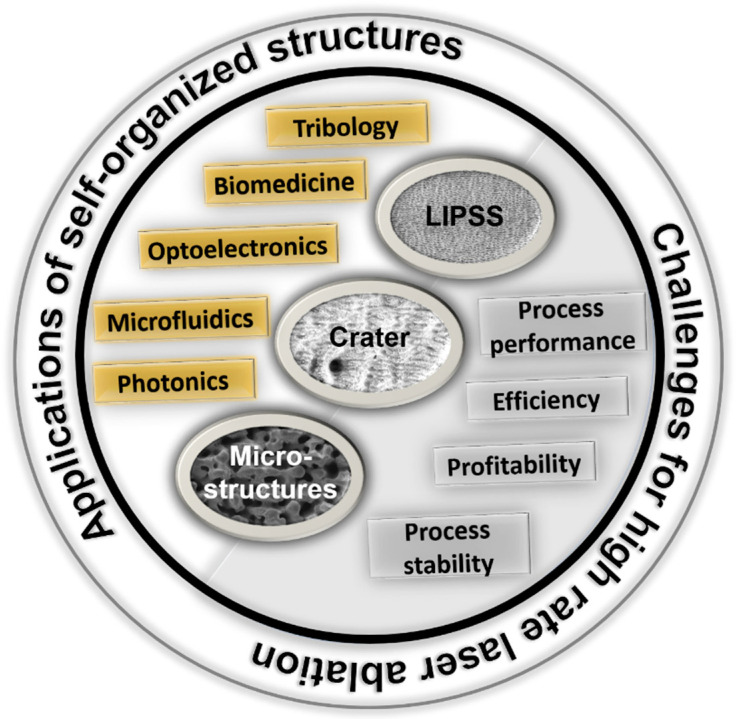
Two strands concerning self-organized structures. On the one hand, LIPSS, crater, and microstructures offer a wide range of technical and biomedical applications. On the other hand, self-organized structures have to be controlled when aiming for smooth surfaces and high surface quality.

The formation of self-organized structures is significant for USPL processing from two points of view, as illustrated in Figure 1. On the one hand, self-organized structures degrade the surface quality and limit laser processing for ablation scenarios aiming for smooth surfaces at simultaneously high ablation rates [17]. In particular, heat accumulation at high pulse repetition rates results in an early formation of craters and microstructures and therefore restricts the scalability of USPL processing [1,3].

On the other hand, it has been shown that self-organized structures offer high potential to tailor optical, chemical, and physical surface properties for technical and biomedical applications [12]. In technical applications, self-organized structures can improve the tribological performance of friction partners [18], modify the reflection and absorption properties of the surface for optoelectronics and solar absorbers [19,20,21,22,23,24], for semiconductors [25,26,27], and enhance the sensitivity of photodetectors [28,29]. In biomedical applications, self-organized nano- and microscaled surface textures can tailor cell adhesion and cell growth, and can be used to control cell proliferation [30,31,32,33,34]. Furthermore, it has been shown that self-organized structures can improve the biocompatibility of materials [35].

The applications mentioned reveal the great potential of self-organized surface structures. At the same time, however, these structures can also be undesirable under certain circumstances and limit the scalability of USPL processing. For both scenarios, intelligent concepts can help monitor and control the formation of self-organized structures. However, such approaches must consider different challenges in material processing.

Firstly, changes to the hardware of laser systems can lead to inconsistent results in surface modification. The quality of the processing results largely depends on the homogeneity and characteristics of the laser beam profile. However, possible changes can occur due to the laser system’s or beam guidance’s instability. Furthermore, the laser crystals used as active media in solid-state lasers age over time, causing their optical properties to deteriorate. In addition, gradual heat-induced warpage of the beam guidance optics can lead to a slight, progressive defocusing of the laser spot in the working area. As a result, USPL processing can lead to irreproducible results or degradation of the surface quality [1,36], even with already-defined parameters. These facts make it necessary to develop quality assurance systems that ensure consistent results in USPL manufacturing.

Second, the formation of self-organized structures is difficult to predict, and transferring laser parameter findings from one material to another is challenging. Currently, costly parameter studies must be performed to realize or avoid self-organized structures on specific materials. This is mainly due to the nature of the formation of self-organized structures, which, as already mentioned, depend on quite complex interactions of laser light with the material surface. Thus, process parameter recommendations based on machine learning (ML) algorithms can simplify and save USPL processing time. Finally, quality assurance systems and process parameter recommendations based on machine learning could be coupled with the micromachining center control to realize inline laser parameter control. This principle would significantly increase the status of USPL processing in manufacturing, but this is still in its infancy.

To date, far too little attention has been paid to ML for the monitoring and automated laser parameter development in USPL processing. However, the advantages of ML for laser processing are increasingly being recognized in the literature. Based on neural networks, real-time feedback process monitoring can be realized, ensuring reliable structuring control. Xie et al. [37] have shown that neural networks can ensure precision in laser drilling using femtosecond lasers and can detect beam displacement and unintended laser beam modifications such as the translation or rotation of the beam. Mills et al. [38] also implemented image-based monitoring of material removal of individual pulses via a neural network for femtosecond laser processing. The authors show that learned neural networks can determine the type of material, laser exposure and number of pulses from image data of single pulse exposures on surfaces. Na et al. [39] used a conditional generative adversarial network and scanning electron microscopy (SEM) images of femtosecond laser-structured surfaces to predict surface morphology depending on the laser parameters. The authors found that the developed models could accurately predict SEM images to reveal surface morphology for unexplored combinations of laser fluence and scanning speed. An interesting paper on surface classification has been published by Wang et al. [40]. The authors used the k-means clustering method to automatically classify the LIPSSs into quality classes based on SEM images. It was highlighted that the presented method, including decision boundary determination, reduces time-consuming and costly trial-and-error laser parameter studies, and can be used to determine an optimized processing window for LIPSS morphology.

The few studies addressing ML algorithms for surface structure modification using USPL processing focus on SEM images to train and test data. However, SEM technology cannot be integrated into USPL machining centers since technology-required vacuum conditions cannot be practically provided. Apart from the very high cost of SEM devices, it is likely that charging effects from primary electrons affect the ablation process. In sum, the methods proposed for SEM images can provide essential theoretical information for USPL processing but will elude practical application in laser micromachining systems.

With the vision for using ML for quality assurance systems, process parameter recommendations, and inline laser parameter control in USPL processing, we present a new ML approach to classify surface structures based on digital light microscope images. In order to evaluate the approach as quickly as possible, an existing open-source algorithm for general classification tasks was used for ML analysis and trained, as well as validated with specially collected data. The complete development of a new ML algorithm from scratch, specifically for the classification of different self-organized structures, was initially dispensed with, as this would have taken considerably more time and also required significantly more training data. As a basis for the training data, significantly different self-organized structure types (LIPSS, CRATER, and MICRO structures) were generated on two different steel substrates (hot work tool steel (HWTS) and stainless steel (SS)) using a 300 fs laser.

The study was divided into four principal workflow steps. First, the different surface structure types and a smooth reference (REF) on the HWTS substrate were imaged using a digital microscope. Second, 250 images per structure class were used to learn a customized algorithm based on the open-source tool Teachable Machine. In this context, the detection accuracy of the developed algorithm was determined by implementing validation samples of the same substrate. Subsequently, the algorithm was tested with test data of both steel substrates to analyze applicability and transferability to different materials. Finally, investigations were carried out to classify the specific structures captured at different optical magnifications to demonstrate the robustness of the proposed method.

## 2. Materials and Methods

### 2.1. Preparation Samples

The hot working tool steel used (X37CrMoV5-1, material designation 1.2343 according to DIN EN ISO 4957 [41]) was purchased from ABRAMS Industries GmbH & Co. KG (Osnabrück, Germany) as plates ((70 × 70 × 5) mm). Tool steel is widely used in molding and tool-making for injection molding machines. Here, surface structures are used to improve molding processes, for example. The specimen plates ((100 × 100 × 1.5) mm) of stainless steel (X5CrNi18-10, material designation 1.4301 according to DIN EN 10088-2 [42] and DIN EN 10088-3 [43]) used for validation were purchased from K&D Handel (Mönchengladbach, Germany). This stainless-steel substrate is used, for example, in the food industry, where nano- and microstructures are relevant for the formation of self-cleaning surfaces. Prior to laser structuring, all samples were ground with a Saphir 520 grinding machine from ATM Qness GmbH (Mammelzen, Germany). A homogeneous surface quality was ensured using sizes 600, 1200, 2400, and 4000 grit sandpaper before investigations were performed. After grinding, the samples were cleaned with isopropanol (purity > 99.5%). The specimens polished and cleaned in this way served as a reference (REF).

### 2.2. Laser Treatment

All laser treatments were performed with a 300 fs fiber laser with an amorphous glass Yb-doped core (UFFL_60_200_1030_SHG from Active Fiber Systems GmbH (Jena, Germany)). A laser wavelength of 1030 nm and a pulse repetition rate of 150 kHz were used for all experiments. The Gaussian laser beam was focused via an F-theta lens with a focal length of 163 mm, leading to a circular focus diameter of d_f_ = 36.6 µm at 1/e^2^ intensity. A constant pulse overlap (PO) of 50% and a line overlap (LO) of 80% were achieved by deflecting the focused laser beam with a scan head of the type intelliSCAN 14 (Scanlab GmbH, Puchheim, Germany) [15]. The fluence was systematically increased to form different types of self-organized structures on the surface [3]. The LIPSSs were generated using a fluence of F = 0.116 J/cm^2^. For the CRATER structures, a fluence of F = 2.011 J/cm^2^ was chosen, and the MICRO structure was generated at a fluence of F = 13.971 J/cm^2^. A constant number of over-scans of N = 50 was applied to achieve the different types of structures. These laser and scanning parameters were used for both steel materials and are based on our previous study [15]. After the laser treatment, the structured samples were cleaned with isopropanol (purity > 99.5%) in an ultrasonic bath for t = 10 min. After polishing and laser processing, confocal laser scanning microscopy (CLSM) measurements with a LEXT OLS 4000 and the software OLS 4000 (Olympus, Hamburg, Germany) were performed to calculate the average area surface roughness Sa (with λc = 25 µm). Scanning electron microscopy images were also taken using a SUPRA 25 field emission scanning electron microscope from Zeiss (Oberkochen, Germany) to evaluate the surface morphology after USPL processing. These images were not used for the ML process.

### 2.3. Machine Learning for Image Recognition and Data Classification

#### 2.3.1. Microscopy, Data Generation, and Data Preprocessing

**Figure 2:**
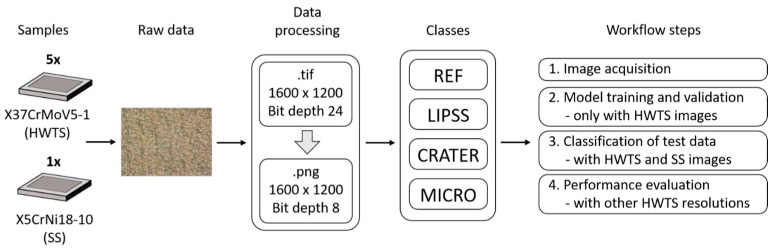
Schematic overview of the data preprocessing and the principal structure of the training and test datasets.

With the perspective of application and integrability into a USPL machining center, we chose a digital light microscope of the type VHX-5000 with VH-Z50 objective (Keyence, Osaka, Japan) to image the sample surface. Based on this technology, four workflow steps were performed, as shown in Figure 2. First, raw data were generated from five HWTS samples and one SS sample to create training and test datasets for the algorithm. An optical magnification of 3000× was used for the LIPSS class images, 2000× for the CRATER class and 1000× for the REF and MICRO classes. The different magnifications were chosen in order to display the individual structural features as detailed and comprehensively as possible. The raw data consist of 1600 × 1200 pixel (px) microscope images in the TIF format for each surface structure type (REF, LIPSS, CRATER, MICRO). Subsequently, each image was compressed using a self-programmed Python script (version 3.7.9, python software foundation, Fredericksburg, VA, USA) and saved as a PNG file. In the second workflow step, the images of all HWTS samples and classes were combined into a training dataset of 1000 images, with 50 images of each structural type taken from each HWTS sample, resulting in a balanced dataset of 250 images per class for training. From this training dataset, approximately 85% of the images were used for training (848 images) and 15% for validating the ML algorithm (152 images). In a third workflow step, 120 additional test images from the five HWTS samples and 120 test images from the SS sample were acquired and merged into two independent test datasets. The trained ML algorithm was tested with these test datasets to verify its classification accuracy and effectiveness on the different steel substrates. In the fourth step, a further performance evaluation was undertaken. Here, the effect of different optical magnifications was investigated to validate the trained algorithm for the classification of surface structure types and test the robustness of the algorithm. For this purpose, five images of all structure classes of one HWTS sample were taken at magnifications of 1000×, 2000×, and 3000×, and tested with the trained algorithm. This allowed us to evaluate whether the ML classification could correctly recognize and classify images with less resolved structural features.

#### 2.3.2. Machine Learning Development and Hyperparameter Tuning

The web-based open-source tool Teachable Machine (https://teachablemachine.withgoogle.com, Google LLC, Mountain View, CA, USA), accessed on 31 January 2023, was used to create an ML model to automatically classify the different structure types. Teachable Machine provides a graphical user interface (GUI) for an easy creation of customized classification models without any particular ML expertise [44]. The Teachable Machine GUI is used to upload the data, set the model parameters, and train the model. The trained model can be exported and used for further implementations, such as web applications.

The accuracy of ML models can be improved by adjusting hyperparameters [45]. Adjusting the appropriate parameters to enhance model accuracy is known as hyperparameter tuning. For Teachable Machine, the following three parameters can be varied to optimize the model:

- Epochs: This specifies how many times each individual image in the training dataset is input to the ML model at least once [46] (i.e., for epochs = 50, the ML model goes through the entire training dataset 50 times during training). More epochs generally allow for better prediction accuracy but increase the risk of overfitting the ML model to the training data [45,46].
- Batch size: This is the size of a set of images used for a training iteration [46] (i.e., for a batch size of 16 and a training dataset of 160 images, the data are divided into 160/16 = 10 batches and entered into the model). Once all stacks are entered, an epoch is completed.
- Learning rate: This determines the step size of the loss function and controls how fast a model adapts to the classification problem [46]. Detailed information about the learning rate can be found in [47].

For the studies in this paper, the following settings were chosen: epochs = 50, batch size = 16, and learning rate = 0.001.

### 2.4. Application Development and Performance Evaluation

Specific performance metrics are used to evaluate the performance of the trained ML model. The model results can be summarized in a classification task as a confusion matrix (CM) [48]. The CM is a crosstab that captures the number of predicted and actual classes that occur [49]. 

Table 1 shows an example CM for these studies. Most important are the true positive (TP) values, which represent the correctly predicted surface structures. True-negative (TN) values correspond to all other misclassified surface structures. False positive (FP) and false negative (FN) values are the sum of the misclassified elements in the columns and rows. They are relevant for the calculation of other performance metrics.

In addition, the accuracy and loss functions are plotted to evaluate the performance of the ML model [50]. The accuracy graph shows the performance of the classification in percentage terms; the loss graph shows the uncertainty of a prediction based on the deviation from the actual value [50,51]. When training the algorithm, the accuracy value should be constantly optimized and the loss value minimized. Both diagrams thus characterize the training process and indicate the effectiveness of the selected hyperparameters [51].

For better interaction with the ML model, a simple web application was also developed and programmed, through which test data can be entered and classified. For this purpose, the data must be stored in a database with a web address or uniform resource locator (URL). The web application was programmed using node.js, HTML5, CSS, and JavaScript. The test data were published via Microsoft Azure Blob Storage (Microsoft, Redmond, WA, USA) which provides a durable and scalable storage service for large amounts of data.

## 3. Results and Discussion

### 3.1. Surface Structure Types

**Figure 3:**
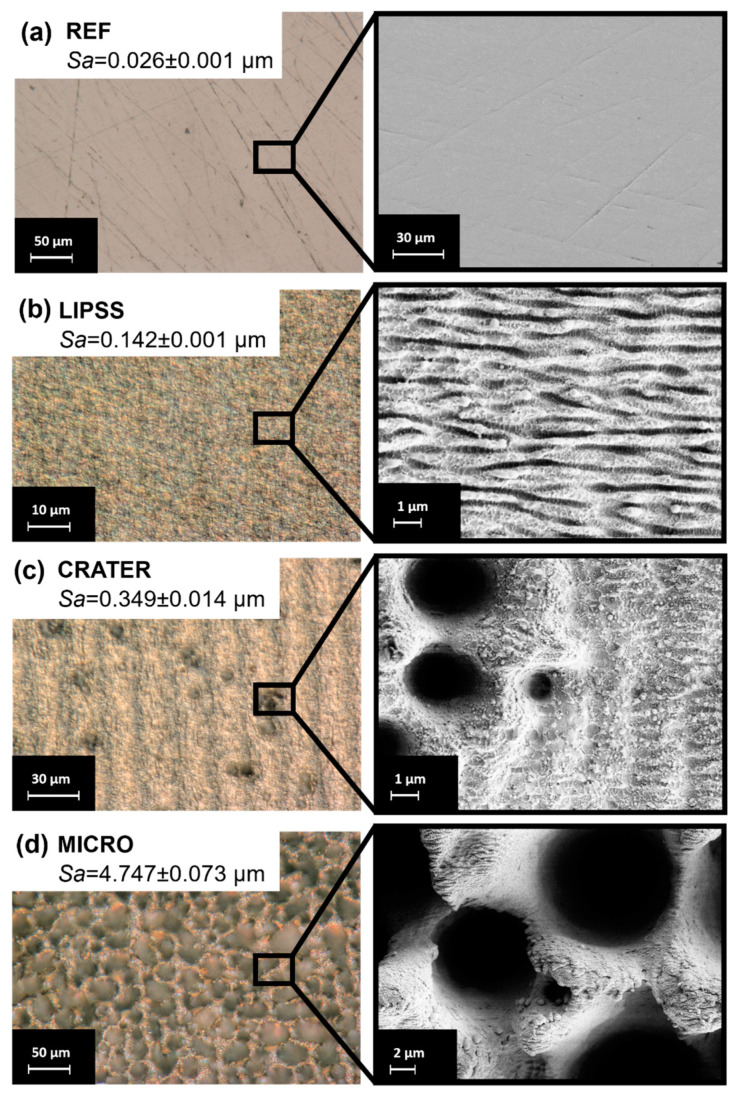
Digital microscopy images, with the calculated average area surface roughness Sa (**left** column), and corresponding SEM images (**right** column) of surfaces after polishing and laser treatment, respectively. (**a**) Reference after polishing (REF); (**b**) nanoscaled roughness of laser-induced periodic surface structures (LIPSSs); (**c**) formation of microscaled depressions (CRATER). Micrometric ripple marked as MR; (**d**) hierarchical roughness of self-organized microstructures (MICRO).

The resulting surface types after the polishing process and laser treatment are shown in Figure 3a–d. The digital light microscopy images and the corresponding SEM images provide qualitative insights into the surface morphology of the different surface classes. More specifically, the surface topography data obtained with CLSM measurements are summarized in Figures S1–S4 in the Supplementary Information. When comparing the different surface types, it is clear that the surfaces obtained significantly differ in surface morphology and topography.

The polished reference (REF) specimens exhibit a homogeneous surface with only marginal scratches from the polishing process (Figure 3a). However, it is clear from the SEM images and average area surface roughness (Sa = 0.026 ± 0.001 µm) that these scratches exhibit a depth in the nanometer range. In the next step, these preprocessed surfaces were laser-treated to create different types of self-organized structures. As can be seen in Figure 3b–d, all laser parameter settings successfully resulted in the formation of different structure types.

First, laser-induced periodic surface structures (LIPSSs) were realized using a fluence of 0.116 J/cm² (Figure 3b). Laser-induced periodic surface structures are wavelike surface structures with a resolution much smaller than the size of the laser spot. It is widely accepted that LIPSSs on metals are mainly formed through electromagnetic interactions between the material surface and the incident laser light. Generally, two different LIPSS morphologies can be distinguished: low-spatial-frequency LIPSS (LSFL) and high-spatial-frequency LIPSS (HSFL). LSFLs typically form on metal surfaces with an orientation perpendicular to the direction of the polarization of the laser light [52]. The spatial periodicity of LSFLs is usually close to the wavelength of the laser beam applied. In contrast, HSFLs exhibit an orientation parallel to the polarization of the incident laser light [52] and have a spatial periodicity much smaller than the laser wavelength [53]. The LIPSS fabricated in this study can be characterized as an LSFL (see height elevation profile in Figure S2 in the Supplementary Information) and are homogenously distributed on the surface. The average area surface roughness is slightly increased (Sa = 0.142 ± 0.001 µm) due to the formation of the LIPSS compared to the nearly smooth reference (REF).

Second, non-homogeneously distributed microscaled depressions, named CRATER, were realized using a laser fluence of 2.011 J/cm² (Figure 3c). The formation of CRATER structures is attributed to local light intensity peaks due to scattering effects on the micrometric ripple [3,54] (marked as MR in Figure 3c). These phenomena lead to a locally preferential ablation on these precursor sites (MR) and consequently to the expansion of the interspaces by the laser shots following. The preferential ablation in the resulting valleys leads to a further deepening of the structures up to the formation of craters [54]. In addition to CRATER and MR, LIPSS with a changed morphology remain present on the surface. These multiscale surface features increased surface roughness (Sa = 0.349 ± 0.014 µm) compared to the REF and LIPSS classes.

Third, MICRO structures were realized at a fluence of 13.971 J/cm², as shown in Figure 3d. MICRO structures are the result of the holistic formation of surface depressions due to an abrupt increase in the ablation rate (known as the “strong ablation phase”) [55]. It is assumed that cone-shaped MICRO structures result from crater propagation, an altered energy distribution due to surface topography during laser irradiation, and build and rebuild effects, e.g., due to the merging of adjacent protrusions [3,54]. However, the average surface roughness is significantly increased compared to the other structure types realized in this study (Sa = 4.747 ± 0.073 µm). Furthermore, the cones are covered with nanoscaled LIPSSs and melt, leading to a hierarchical structure.

Overall, the processing methods resulted in significant differences in surface morphology and topography. These surface features lead to different surface optics due to varying levels of light adsorption and reflection under illumination during microscopy. Therefore, the captured images reflected these surface features and formed the basis for this intelligent approach to surface classification (first workflow step).

### 3.2. Machine Learning Model Implementation

**Figure 4:**
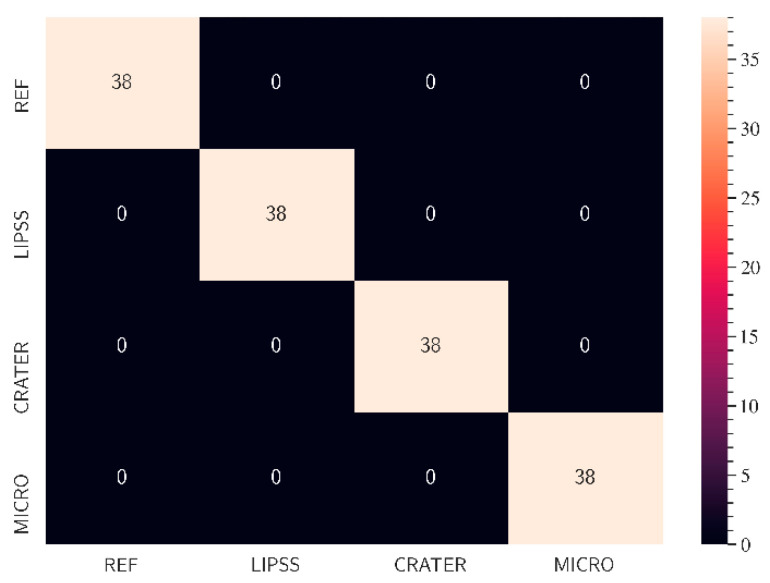
Results of ML classification with the validation data of the training dataset in the form of a CM (second workflow step).

In the second workflow step, an ML model with customized settings was defined via TeachableMachine and trained with the created training dataset. The results of ML-based classification of light microscopy images of the different surface structures based on the training dataset are presented in Figure 4, which illustrates the performance of the ML model in a CM. The model can correctly distinguish between all analyzed images and classify the validation images into their corresponding classes. Therefore, the accuracy of the algorithm is 100% for all surface classes.

**Figure 5:**
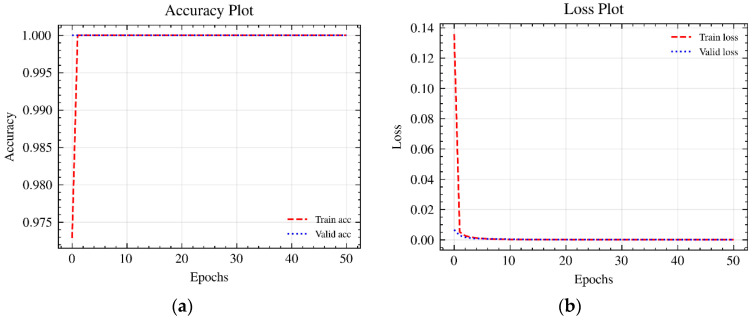
Accuracy (**a**) and loss plots (**b**) of the training and validation data.

To check the plausibility of the result, the accuracy and loss plots of the ML model were generated. Figure 5a shows the accuracy curves of the training and validation data. The Teachable Machine ML model used about 85% of all training data for training and about 15% to validate the trained model. The validation data were randomly selected and were not used to train the model but only to perform an initial performance evaluation of the model using data that are unknown to it [46]. Figure 5b shows the loss curves of the training and validation data.

The accuracy and loss curves all show optimal behavior. The accuracy of the ML model increased and reached 100% after just one epoch. At the same time, the loss curves continuously decreased to almost zero across all epochs. The validation data confirm this trend in every case. The results are comprehensible since the images of the individual structure types used for training look distinctly different and each has characteristic morphological features. The different surface structure features on the light microscopy images are visible to the naked eye. The ML algorithm also searches for the characteristic image features during the training process and finds them very quickly. As a result, the algorithm can distinguish the different structure–type images very well. This happens automatically after the training process without human influence so that the ML model can function as part of an automated quality assurance process. However, it should be noted that the performance of the ML model is currently based on training with a comparatively small and poorly differentiated training dataset. The images corresponding to this dataset were all taken under laboratory conditions and are relatively similar. Furthermore, the results are based only on images from the digital light microscope, which was used to capture surface structures on just one material. Different results are expected for images that deviate from optimized conditions (e.g., lighting conditions, sharpness, color values, etc.) or were acquired via other systems (e.g., microscopes with a lower resolution) or of other materials. To achieve optimal results with the ML model for a wide range of different image conditions, materials or systems, the algorithm needs to be more robust, e.g., by training it on more differentiated data. In addition, it would be a suitable development if the algorithm were to directly highlight relevant surface features. This would help better understand the underlying basis for the classification used by the algorithm. In this regard, the ML algorithm needs further development to detect different surface features in addition to classification. This could be realized by gradient-weighted class activation mapping (GRAD-CAM) [56] or by you only look once (YOLO) detection algorithms [57,58,59].

By linking the surface structure images with the respective laser parameters, the ML algorithm can also be extended for process parameter recommendations. In this context, the ML algorithm could determine which laser parameters were used and how they can be optimized if necessary. Thus, the ML evolves from a classification over a detection to a decision-making and recommendation system. This can ultimately be integrated into a comprehensive hardware, software, and data system. This enables the monitoring of laser structuring and closed-loop processes in the sense of an inline process.

**Figure 6:**
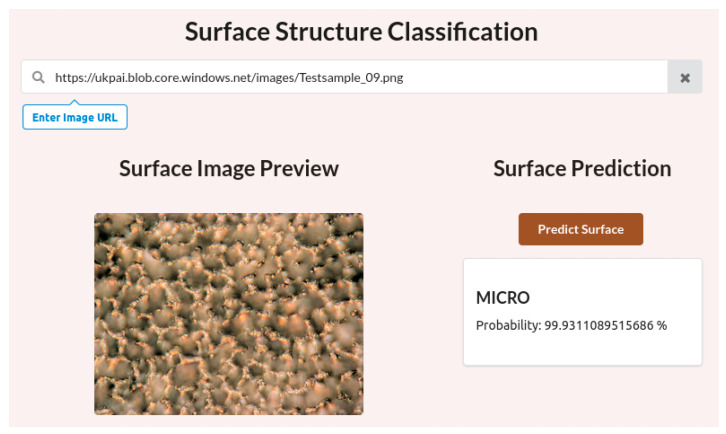
Web application for surface structure classification.

In summary, the ML model trained with Teachable Machine initially enables the classification of the surface structures. The intended classification task is already perfectly trained with the selected hyperparameters and the training data used are suitable, for example, for integration into a web application to analyze further test data. An example of a GUI web application is shown in Figure 6. This is intended to represent a possible quality assurance application as it might be used in practice.

Via the web application, all images published online can be searched via their respective URL and displayed in the image preview. The “Predict surface” button can be used to start an image analysis with the trained ML algorithm. As a result, the present surface structure is classified with associated probability. In this way, the examined structure is quickly classified automatically which can be used to establish automated quality assurance processes.

**Figure 7:**
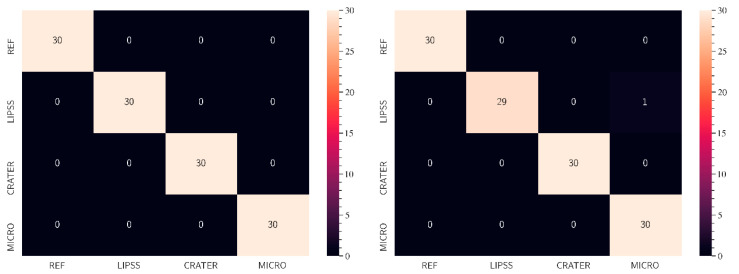
CM of the test datasets. **Left**: evaluation of the HWTS sample results; **Right**: evaluation of the SS sample results (third workflow step).

All images of the two test datasets (HWTS and SS) were analyzed via the developed web application (third workflow step) for a detailed evaluation of the trained ML algorithm. With the web application and the trained ML algorithm, all test data of the HWTS samples could be classified correctly (see Figure 7, left), which was to be expected since the algorithm was trained with very similar data. The test data of the SS sample could also be distinguished especially well, but here, the algorithm shows weaknesses in the recognition of LIPSS structures (see Figure 7, right). In one case, a LIPSS structure was incorrectly detected as a CRATER. There are several reasons for this slight inaccuracy in the prediction. First, the acquisition conditions of the images of the SS sample are slightly different from those of the HWTS samples (the images are brighter, for example). Second, some LIPSS structures show darker areas, which could also indicate CRATER for the algorithm. Ultimately, this finding suggests that the algorithm still needs robust training. The training dataset would need to include more differentiated images to reduce the influence of different imaging conditions. Accordingly, data from different process configurations, materials, and image representations should be included in the training of the ML model.

**Figure 8:**
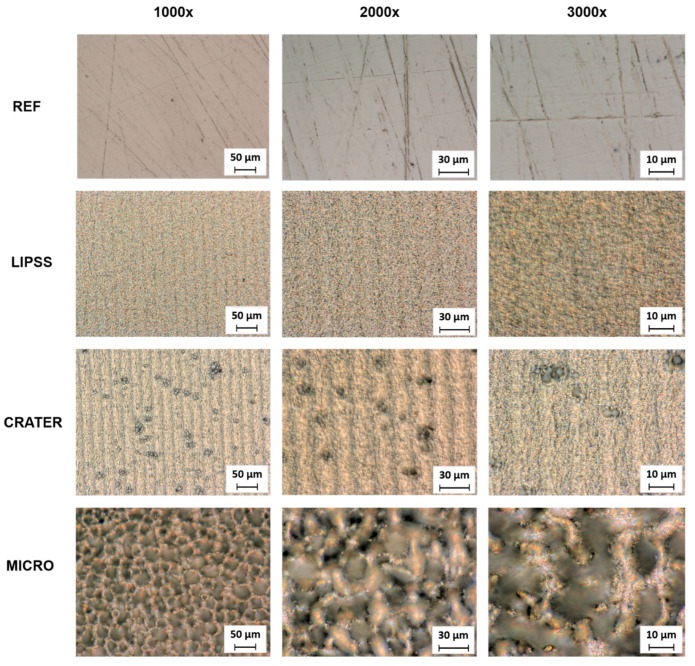
Overview of the surface structures captured at different magnifications.

**Figure 9:**
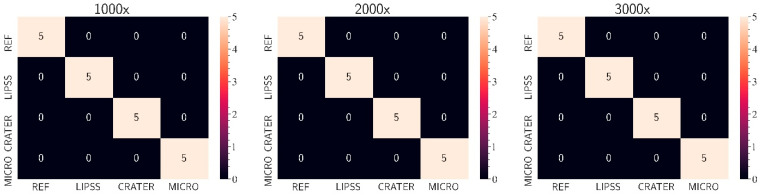
CM of the tests with different magnifications (fourth workflow step).

To evaluate the accuracy and robustness of the algorithm with less optimal images, images captured at different magnifications of the individual structure types were tested via the web application (see Figure 8). As can be concluded from Figure 9 (fourth workflow step), the algorithm is also suitable for classifying blurred and low-resolution images. This means that the training method used (with images of one magnification within one structure type but different magnifications between the individual structure types) enables the algorithm to classify images with varying resolutions correctly and very effectively. Thus, although it was trained with a small training dataset, the algorithm is already relatively robust. Furthermore, high-resolution images are optional for reliable classification.

## 4. Conclusions

In this study, a new approach for classifying laser-induced surface structures with an ML based on digital light microscopy images was developed and evaluated. The findings show that a customized algorithm based on the open-source tool Teachable Machines is highly suitable for classifying self-organized structures resulting from USPL processing. The special feature of the classification algorithm presented here is that it already works in a very stable way, even with a small amount of training data. After short learning times, reliable classifications could be achieved regardless of the different magnifications used for microscopy. Furthermore, the findings show that the algorithm provides highly accurate results for similar substrates to those learned. In contrast to the few studies conducted so far on surface classification in USPL processing using SEM images for training data, this approach can be integrated into a micromachining center. Therefore, these findings lay the groundwork for quality assurance concepts, process parameter recommendations, and inline laser parameter control for USPL processing.

However, further research is required to further develop this concept for laser parameter recommendation systems and for integration into laser processing systems, e.g., for inline process control. Future work should link the process parameters used with surface classes and topography data. This will form the basis for predicting surface features using ML. It is also recommended to acquire more data from different materials, laser processing studies, and light microscopy images with a variety of setups and conditions to improve the robustness of the algorithm. Furthermore, the web application presented in this study should be extended to include direct interfaces for the microscope. This would enable direct image analysis while laser-structuring the surfaces. Lastly, data pre-processing should also be optimized in order to generate image files that are as small as possible but sufficiently detailed for analysis. This can reduce the inconsiderable memory requirements as image data continue to increase, thus saving costs.

## Figures and Tables

**Table 1:** Principal CM scheme for the evaluation of the microstructure class predictions.

		**Predicted Class**				
	Classes	REF	LIPSS	CRATER	MICRO	total
**True Class**	REF	TP	TN	TN	TN	FN
	LIPSS	TN	TP	TN	TN	FN
	CRATER	TN	TN	TP	TN	FN
	MICRO	TN	TN	TN	TP	FN
	total	FP	FP	FP	FP	

## Data Availability

The data presented in this study are available on request from the corresponding author. The data are not publicly available due to privacy.

## Supplementary Materials

The following supporting information can be downloaded at: https://www.mdpi.com/article/10.3390/mi15040491/s1, Figure S1: Height profile of the REF sample with details; Figure S2: Height profile of the LIPSS sample with details. Measurement was performed perpendicular to the orientation of the LIPSS; Figure S3: Height profile of the CRATER sample; Figure S4: Height profile of the MICRO sample.
