# Supplementary material for: Machine Learning Classification of Self-Organized Surface Structures in Ultrashort-Pulse Laser Processing Based on Light Microscopic Images
Source: Micromachines (Basel). 2024 Apr 2;15(4):491. doi: 10.3390/mi15040491 (PMC11051940; doi:10.3390/mi15040491)
Supplement: Supplementary file 1 [file micromachines-15-00491-s001.zip › micromachines-2918783-supplementary.pdf]

Supplementary Materials

Hight elevation profiles for surface structure types (REF, LIPSS, CRATER, MICRO).

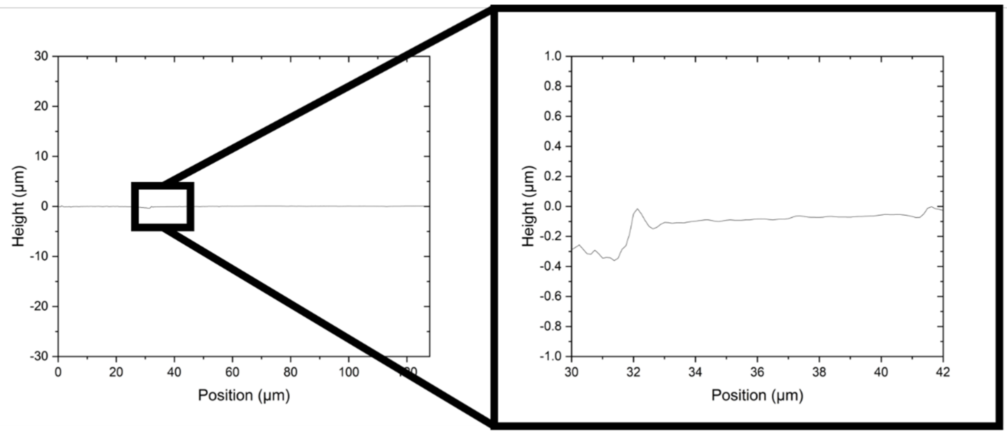

Figure S1. Height profile of REF sample with detail.

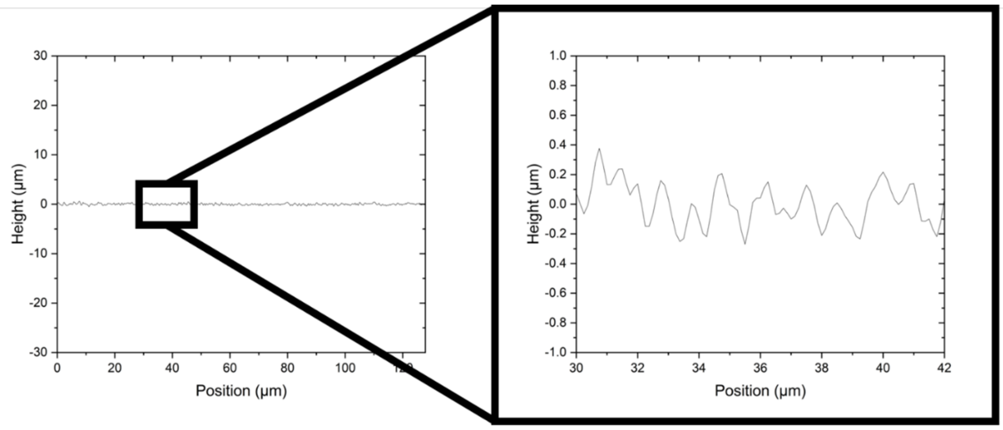

Figure S2. Height profile of LIPSS sample with detail. Measurement was performed perpendicular to the orientation of the LIPSS.

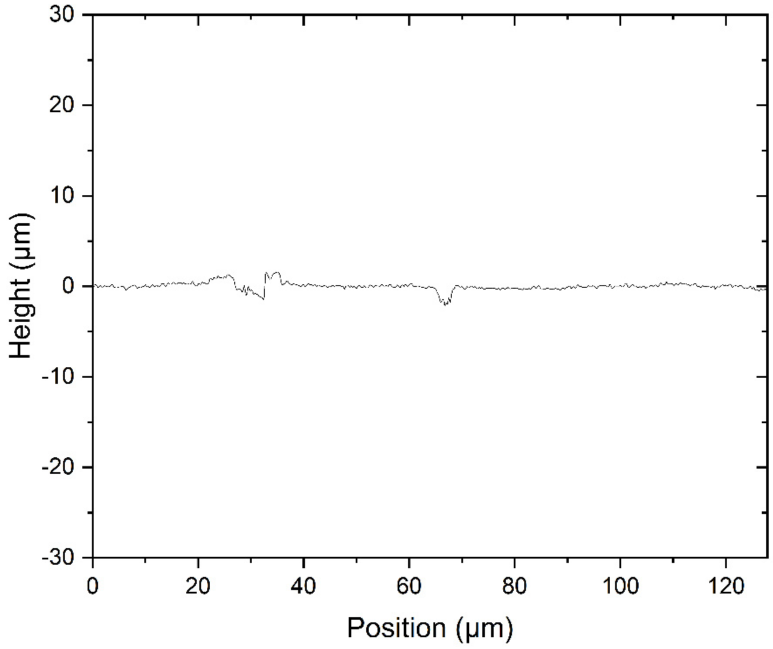

Figure S3. Height profile of CRATER sample.

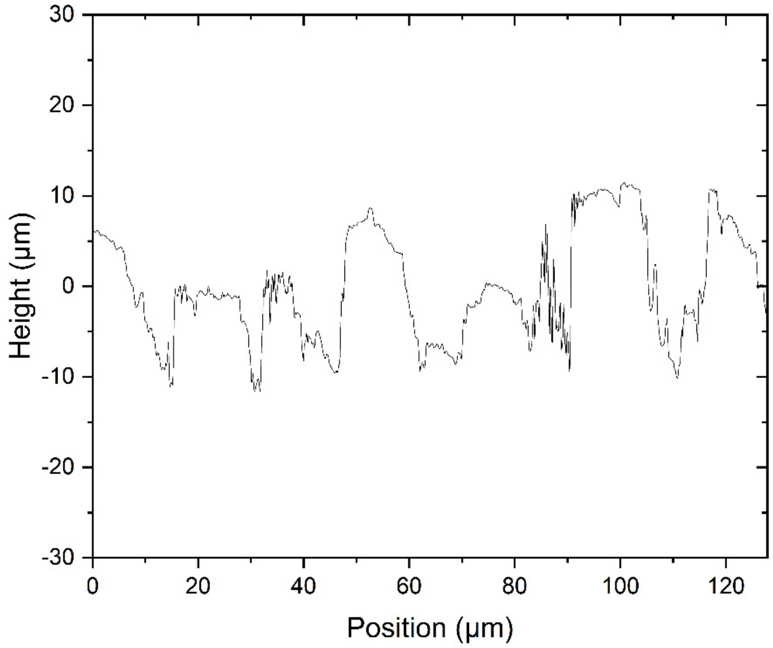

Figure S4. Height profile of MICRO sample.
